# Effects of topical atropine on intraocular pressure and myopia progression: a prospective comparative study

**DOI:** 10.1186/s12886-016-0297-y

**Published:** 2016-07-19

**Authors:** Chia-Yi Lee, Chi-Chin Sun, Yi-Fang Lin, Ken-Kuo Lin

**Affiliations:** Department of Medicine, Chang Gung University, College of Medicine, 259, Wenhua 1st Rd., Guishan Dist., Taoyuan City, 33302 Taiwan; Department of Ophthalmology, Keelung Chang Gung Memorial Hospital, 222, Maijin Road, Keelung, 20402 Taiwan; Department of Chinese Medicine, Chang Gung University, 259, Wenhua 1st Rd., Guishan Dist., Taoyuan City, 33302 Taiwan; Department of Ophthalmology, Linkou Chang Gung Memorial Hospital, 5 Fuxing Street, Guishan District, Taoyuan, 33305 Taiwan

**Keywords:** Atropine, Myopia, Intraocular pressure

## Abstract

**Background:**

Myopia-related maculopathy is one of the leading causes of blindness in the world. The prevalence of myopia has been reported as high as 90 % in some Asian countries. Therefore, controlling myopia progression is an urgent public issue. The purpose of this study is to evaluate the effects of topical atropine with different concentrations on intraocular pressure measurements and myopia progression in school-aged children in Taiwan.

**Methods:**

Fifty-six myopic children were divided into three groups: 32 children were treated with 0.125 % atropine eyedrop; 12 of them were treated with 0.25 % atropine eye drop and another 12 served as a control group. IOP, auto-refractor and manifest refraction were measured at baseline and every 3 months following treatment for one year.

**Results:**

There were no significant differences for the mean age, gender and baseline IOPs among the three groups. During the follow up period, no significant IOP difference was found among three groups. The change between final and baseline mean IOPs also revealed no significant differences: 0.54 mmHg, −1.28 mmHg, −0.33 mmHg for the 0.125 % atropine, 0.25 % atropine and control groups. The baseline mean spherical equivalent similarly did not differ significantly among groups but the control group showed a significant myopic progression compared to the 0.125 % atropine group 6 months after treatment, and persisted for one year. The change between final and baseline mean spherical equivalents were −0.05 D, 0 D, −1.05 D for the 0.125 % atropine, 0.25 % atropine and control groups, with both atropine-treated groups showing significant myopic retardation compared to the control group.

**Conclusions:**

Topical use of low concentration atropine for one year does not induce ocular hypertension and is effective for retarding myopic progression. However, further large scale studies with longer follow up period is necessary to validate the long term safety and efficacy.

**Trial registration:**

ISRCTN33002849, 2016/01/19, retrospectively registered.

## Background

Myopia is a disease that was described two thousand years ago, and some physicians in Italy in the 16th century even started to use bifocal lenses as a tool to help people with myopia to see more clearly [1]. Currently, multiple interventions or management are designed to control the progression of myopia. Common effective methods for retarding myopic progression include spectacles (bifocals and multi-focal), ocular hypotensive medications, and contact lenses (including orthokeratology lenses) [1–3]. Atropine, a muscarinic receptor antagonist, has been successfully used to prevent progression of myopia in Taiwan since 1997 [4] and several clinical trials also demonstrated its effectiveness for reducing myopic progression compared with other medications and managements [1, 5–9]. In fact, topical atropine has become a mainstream of myopic treatment throughout eastern and south-eastern Asia [1, 5, 6, 8, 9].

However, some people suspect that the side effects caused by atropine will harm eyes. The side effects of topical atropine can be divided into two types: short-term and long-term effects. The short-term side effects include red eyes, photophobia, blurred vision, allergic dermatitis, risk of increase intraocular pressure (IOP) and angle closure glaucoma [1, 10–12]. The development of drug-induced acute angle closure glaucoma (AACG) by atropine was reported by Lachker et al. [10] but Greensteinet al. [13] pointed out that anti-muscarinic drugs such as atropine will not contribute to AACG unless there are predisposing factors such as a shallow anterior chamber and pupil-dilating medications. However, whether atropine leads to elevated intraocular pressure remains uncertain. The long-term side effects of atropine is also a mystery, though some proposed that prolonged ultraviolet light exposure due to the mydriatic effect of atropine could lead to several types of ocular damage, such as retinal vascular disease and cataract formation [14, 15]. In addition, previous reports also demonstrated that atropine may lead to drug-induced amnesia, impaired memory function, somnolence and seizure [16–18] so long-term administration should be cautious. Yet cataract or retinal diseases has not been proven in previous studies which continuously applied atropine with a follow up of 2 years [5, 12]. However, the safety profile of long-term atropine administration certainly deserves large scale and long term surveys.

Still, the relationship between the concentration of atropine and its effect on retarding myopia has not been elucidated. One study revealed that topical atropine with 1 % concentration is an effective method to prevent myopic progression; [6] however, other studies demonstrated that myopia could be successfully retarded with low concentration atropine eye drops, ranging from 0.01 % to 0.1 % [19, 20]. For these reasons, further evaluation of the effect of different concentrations of topical atropine on myopia progression is necessary.

Therefore, the aim of this study is to evaluate the effect of different concentrations of topical atropine on retarding the progression of low myopia and the measurements of intraocular pressures in school-aged children in Taiwan.

## Methods

### Patient group selection

This prospective, interventional, longitudinal and non-randomized study was conducted at Keelung Chang Gung Memorial Hospital, Taiwan. The study group consisted of 56 children in Northern Taiwan ranging in age from 6 to 12 years. The exclusion criteria included: [1] congenital eye disorder, [2] any disease influence the cornea, lens or retina, [3] best correct visual acuity < 20/25 using the Snellen chart, [4] primary intraocular pressure above 21 mmHg, [5] atropine application within 6 months before enrollment, and [6] patients who could understand the details of this study or could not adhere to the follow up schedule. Patients with a refractive error less than −3.0 diopters (D) were enrolled in this study and only the right eye of each subject was analyzed to prevent effect of atropine on the contra-lateral eye. For those participants who were willing to receive atropine treatment, we randomly assigned them to either 0.125 % atropine group or 0.25 % atropine group by drawing lots. Those who preferred spectacles for correcting myopia were enrolled in the control group without any eyedrop use during the study. After removing those participants that loss follow-up or being absent in at least 1 clinic visiting, we divided the participants into three groups. The first group, treated with 0.125 % atropine, consisted of 32 children (17 boys and 15 girls); the second group using 0.25 % atropine included 12 children (4 boys and 8 girls); and an additional 12 children (6 boys and 6 girls) in the control group. The intraocular pressures and spherical equivalent were measured every 3 months during the study period for one year.

### Atropine administration

Two topical atropine sulphate eye drops were used in this study: 0.125 % and 0.25 % (both from Wu-Fu Laboratories Co, Ltd, Yilan, Taiwan). The concentration we used was the original concentration from the pharmacological corporation. No dilution or condensation happened during the whole study and no patient shared the same bottle of atropine. The research program was halted if severe allergic reaction happened to the patients, and the proper management was subsequently arranged by medical professionals. The control group did not receive any placebo eye drop in this study.

### Ocular examination

All children were examined by the same technician (Y. F. Lin) for the measurement of intraocular pressure and the manifest refraction examinations during the study period. The slit-lamp biomicroscopy and fundoscopic examinations revealed normal status except for the refractive errors before the study. Because of rapid measurement and a good correlation with Goldmann applanation tonometer [21], we used a pneumatic tonometer (NT-530P, Nidek Co., Ltd, Gamagori, Japan) to measure the IOP in our patients. Each patient received 3 measurements and the average of those measurements was the final IOP. The spherical equivalent was measured and calculated by a Topcon auto-keratorefractometer (KR-3000, Topcon, Yamagata, Japan). All the refractions were performed under cycloplegic condition. If there was an elevation of IOP more than 5 mmHg compared to baseline, severe allergic reactions or other ocular side effects, we immediately halted the atropine application and withdrew the patient from this study. Our subject wore spectacle and no contact lens, progressive or photochromic lenses were applied during the whole follow up period.

### Statistical analysis

All statistical analyses were conducted using SPSS software version 19 (SPSS Inc., Chicago, Illinois, USA). The gender and age among groups were evaluated by the Chi-square test and One-way ANOVA sequentially. For the comparisons of IOP and refractive errors within each group during different time periods, the pair-t test was applied. While comparing the IOP as well as refractive errors among the three groups, we analyzed the variance first. If the variances among the three groups were homogenous, we used One-way ANOVA for analysis and added Bonferroni test for post-hoc analysis. But if the variances among the three groups were heterogenous, we used Welch’s test for analysis and added Dunnett T3 test for post-hoc analysis. Confidence intervals of 95 % were regarded as a comparison for mean values and *p* < 0.05 was considered statistically significant in pair-t test, Chi-square, One-way ANOVA and Welch’s test but <0.025 in Bonferroni test and Dunnett T3 test since we need to reduce the family-wise error in post-hoc exam.

## Results

**Fig. 1:**
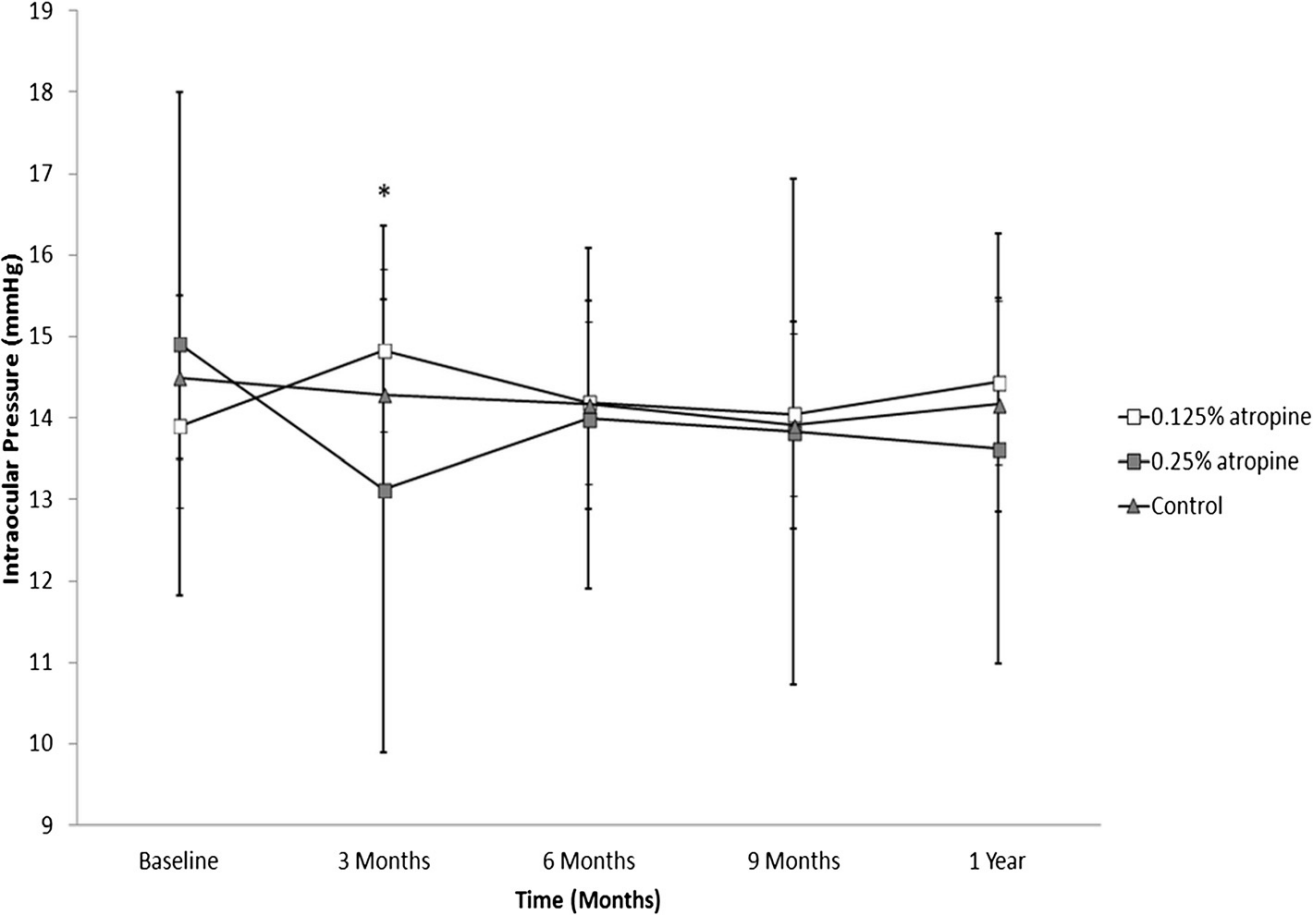
Mean intraocular pressure (IOP) in the three groups during the follow up period. *denotes significant difference compared to baseline mean IOP in the 0.25 % atropine group

The mean age was 9.03, 8.16 and 8.33 years for the 0.125 % atropine, 0.25 % atropine and control groups, respectively. There was no statistical significance for both gender and age (*p* = 0.50 and 0.37, Chi-square test and One-way ANOVA sequentially) among the three groups. The baseline mean intraocular pressure initially was 13.90 ± 3.26 mmHg in the 0.125 % atropine group, 14.91 ± 3.09 mmHg in the 0.25 % atropine group and 14.50 ± 2.43 mmHg in the control group, respectively. The mean IOP measurements and the statistical analyses among the groups during the follow up period are shown in Table 1. All three groups demonstrated no significant difference between group comparisons at each time point during the study period (Table 1). In addition, with the exception of the 0.25 % atropine group which showed a significant difference (*p* = 0.03) at 3 months, all three groups during the follow up period demonstrated no statistically significant difference as compared to their baseline IOPs (Fig. 1). The data, demonstrated that a low concentration of atropine use for one year in school-aged children for myopia control did not induce IOP elevation.

**Table 1:** Intraocular pressure measurements and analyses over one year follow up

	Group			
	0.125 % Atropine	0.25 Atropine	Control	
	Mean ± SD (mmHg)	Mean ± SD (mmHg)	Mean ± SD (mmHg)	*p*
Baseline	13.90 ± 3.26	14.91 ± 3.09	14.50 ± 2.43	0.60
3 Months	14.83 ± 2.43	13.13 ± 3.23^a^	14.29 ± 2.66	0.18
6 Months	14.19 ± 2.81	14.00 ± 2.09	14.17 ± 2.41	0.98
9 Months	14.05 ± 2.40	13.83 ± 3.10	13.92 ± 2.40	0.97
1 Year	14.44 ± 2.54	13.63 ± 2.64	14.17 ± 2.89	0.66

*SD* Standard deviation

*p* = analysis of variance among groups with One-way ANOVA, *p* < 0.05 demonstrates significant difference among three groups.

^a^Significant difference compared to baseline mean IOP was found in the 0.25 % atropine group (*p* = 0.03), analyzed with the paired t test.

**Fig. 2:**
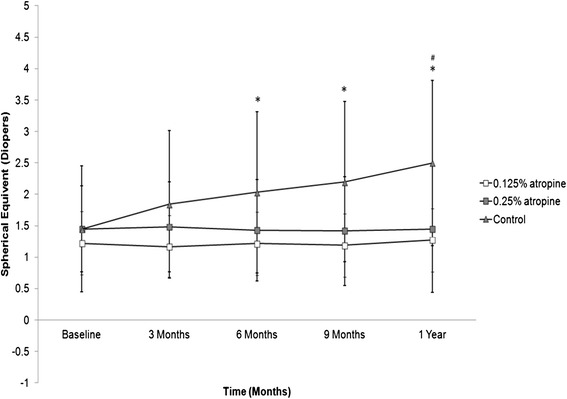
Mean spherical equivalent in the three groups during the follow up period. * indicates significant difference between 0.125 % atropine group and control group; ^#^ demonstrates significant difference between 0.25 % atropine group and control group

The baseline mean spherical equivalent was −1.22 ± 0.55 D in the 0.125 % atropine group, −1.45 ± 0.69 D in the 0.25 % atropine group and −1.45 ± 1.00 D in the control group, which means none of the groups significantly differed from the others (*p* > 0.05). The mean spherical equivalents and the statistical analyses among the groups during the follow up period are presented in Table 2. The mean progression of myopia, i.e., the change of spherical equivalent, was −0.05 D in the 0.125 % atropine group, 0 D in the 0.25 % atropine group and 1.05 D in the control group. We found that the spherical equivalents in the atropine groups remained stable (no significant difference either between 0.125 % and 0.25 % atropine groups or compared to their baseline spherical equivalent at any time point). However, significant progression of myopia was noted in the control group compared to the 0.125 % atropine group (from 6-month after treatment till one year) and to the 0.25 % group (one year) (Fig. 2, *p* < 0.05). Moreover, a significant difference in spherical equivalent among three groups was also noted from 6 months to 12 months with the final refractive errors of −1.27 ± 0.85 D in the 0.125 % atropine group, −1.45 ± 1.00 D in the 0.25 % atropine group and −2.50 ± 1.31 D in the control group after treatment (Table 2, *p* = 0.03, <0.01 and <0.01, respectively, One-way ANOVA), indicating that atropine treatment effectively controlled myopia progression.

**Table 2:** Mean spherical equivalent in each group before and after atropine treatment

	Group			
	0.125 % Atropine	0.25 Atropine	Control	
	Mean ± SD (D)	Mean ± SD (D)	Mean ± SD (D)	*p*
Baseline	−1.22 ± 0.55	−1.45 ± 0.69	−1.45 ± 1.00	0.52
3 Months	−1.16 ± 0.63	−1.48 ± 0.72	−1.85 ± 1.17^Ø^	0.13
6 Months	−1.22 ± 0.70	−1.43 ± 0.80	−2.03 ± 1.28^Ø^	0.03^a^
9 Months	−1.19 ± 0.80	−1.42 ± 0.86	−2.20 ± 1.27^Ø^	<0.01^a^
1 Year	−1.27 ± 0.85	−1.45 ± 1.00	−2.50 ± 1.31^Ø^	<0.01^a^

*D* Diopter, *SD* Standard deviation

Analyses of variance among the three groups were performed with the One-way ANOVA and Welch’s test, ^a^ indicates significant difference among three groups (*p* < 0.05)

Analyses of variance from baseline within the group were performed with the paired t test; ^Ø^Significant difference from baseline spherical equivalent (*p* < 0.05)

## Discussion

Myopia is one of the most common causes of low vision in the world [22] and Chinese children and other Asian populations are more vulnerable to developing myopia, as compared to white populations, especially those who live in urban regions [22–24]. In Taiwan, the myopic group accounts for a large percentage of school-age children, similar to the Chinese population [22, 25]. The anti-accommodation property of atropine was believed to retard myopic progression [26], but it has recently been demonstrated that atropine retards myopia mainly via a non-accommodative mechanism by regulating the muscarinic receptor of the retina, choroid and sclera tissues [27–32]. Accumulating evidence has shown that topical atropine is effective in retarding myopic progression [1, 5, 8, 12, 33]. Kennedy et al. [34] revealed that mean myopic progression during atropine treatment was 0.05 D per year, whereas the control group had an annual progression of 0.36 D. Moreover, Chua et al. concluded that atropine not only retards the progression of myopia but also reduces the axial length elongation [5]. In our study, the mean progression of myopia evidenced no significant difference compared to the baseline in the 0.125 % and 0.25 % atropine groups, though the control group showed a significant progression (*p* < 0.01) during the follow up period. Our study is consistent with others, implying that atropine treatment may prevent myopia progression.

It was also demonstrated that a higher concentration of atropine use results in better myopia control [5, 6, 8, 35] Fan et al. used 1 % atropine and found that progression of myopia was +0.06 ± 0.79 D in the treatment group versus-1.19 ± 2.48 D in control group per year [6]. Chua et al. also reported a 0.92 D difference in myopic progression between the treatment group (1 % atropine) and control group [5]. In addition, Song et al. demonstrated that high concentrations (0.5 % and 1 %) were more effective to retard myopia progression than lower concentrations (0.05 %, 0.1 % and 0.25 %) [8]. A similar study by Shih et al. showed that 61 % of patient in the 0.5 % atropine group had no myopia progression, compared to 49 % in 0.25 % atropine group and 42 % in 0.1 % atropine group, thus indicating that higher concentrations are more effective [35]. However, side effects such as mydriasis, photophobia, blurred vision and allergic dermatitis by high concentration atropine have been reported [1, 7] which may limit the medication compliance [35] and impose psychological burdens on the parents. Cooper et al. demonstrated that 0.02 % atropine is the maximum dose that would not induce clinical symptoms [36], and for this reason, low atropine dose should probably be considered so as to limit adverse effects. In addition, lower concentrations of atropine may also effectively retard myopia progression even at a concentration of 0.01 % [20]. In our study, we found no significant difference between the 0.125 % atropine and 0.25 % atropine groups (*p* > 0.025) with a mean myopic progression of −0.05D in the 0.125 % atropine group and 0D in 0.25 % atropine group for the one year follow up period. Taken together, we recommend that a low concentration atropine should be considered first in terms of retarding myopia progression and avoiding intolerable side effects.

One of the controversial side effects of atropine is its anti-cholinergic effect that leads to drug-induced acute angle closure glaucoma [10]. Although Herring et al. [37] found an IOP decrease in horse eyes after 1 % topical atropine use, Stadtbaumer [38] observed an IOP elevation in feline eyes from the same medication. The same inconsistency was also noted in human eyes. Harris et al. showed that atropine may lead to the elevations of IOP up to 23 % in proven open-angle glaucoma, but only 2 % in an apparently normal population [39]. Hadjikoutis et al. suggested careful use of atropine in neurological operation to prevent elevated IOP and angle closure glaucoma in susceptible patients [40]. However, the threshold concentration that may lead to such side effects was not disclosed in these studies. In a large scale study, Wu et al. [41] analyzed 621 myopic children with atropine dosages ranging from 0.1 % to 1 % and concluded that neither the duration of treatment nor cumulative dose of atropine would statistically elevate IOP. In our study, there was no IOP elevation in atropine treatment groups compared to either baseline measurements or to control group during the one year follow up. Though our results were consistent with those of Wu et al., our study was prospective-designed while their study was retrospective in nature. In addition, we used the average data of three measurements from every visit compared to only one digital reading by Wu et al., which may have increased their measurement errors. Therefore, these studies on school-aged children do not support the linkage between atropine use and the elevation of IOP in normal eyes.

It would be interesting to know if there is any relationship between intraocular pressure and myopia progression. Jensen et al. [42] concluded that IOP-lowering eye drops demonstrated no effectiveness on retarding myopic progression and indicated that IOP has no significant relation with myopia progression. However, a later study conducted by the same team revealed that if IOP was above 16 mmHg, a statistically significant difference of myopia progression would appear (1.32D in 2 years versus 0.86D in 2 years) [43]. The authors thus suggested using IOP measurements in studying myopia progression. A more recently study revealed that IOP would elevate after onset of myopia [44], though the relationship between IOP and refractive error remain unclear [45]. Our study did not evidence the relationship between IOP elevation and myopic progression in one year period, which probably because myopia progression was reduced in the 0.25 % and 0.125 % atropine groups. However, one should keep in mind that myopia may lead to IOP elevation and current study only showed that atropine didn’t contribute to IOP elevation. Therefore, further long-term study is mandatory to investigate the incidence and risk factors of myopic-induced glaucoma.

There are some limitations in our study. First, this study is not a double-blind randomized design. Second, the small sample size and relatively short follow up time are noted in our study. Long term side effects may be apparent in a long period of follow up.

## Conclusions

In conclusion, when it comes to the elevation of intraocular pressure, there was no evidence that ocular hypertension would be increased by the application of atropine for one year in this study. Similar to other studies, topical atropine eye drop is effective in slowing the progression of low to moderate myopia even with a low concentration of 0.125 %. Therefore, low concentration atropine may be used in clinical practice to retard myopia. However, randomized controlled clinical trials with a large sample size and long follow up should be conducted in the future to validate our results.

## Abbreviations

IOP, intraocular pressure; AACG, acute angle closure glaucoma; D, Diopter; SD, Standard deviation; GH, growth hormone
